# The role of radiological imaging in differentiating malignant and benign pulmonary nodules: a retrospective study

**DOI:** 10.1186/s12880-026-02346-8

**Published:** 2026-04-15

**Authors:** Lingbo Deng, Licheng Qiu, Jiao Li, Shuang Wu, Yulin Li, Wen Zhou, Guanxun Cheng

**Affiliations:** 1 Department of Medical Imaging, Peking University Shenzhen Hospital, Shenzhen, Guangdong 518036 China; 2 Department of Pathology, Peking University Shenzhen Hospital, Shenzhen, Guangdong 518036 China

**Keywords:** Pulmonary nodule, Computed tomography, Differential diagnosis, Lung cancer, Diagnostic imaging

## Abstract

**Background:**

Accurate differentiation between malignant and benign pulmonary nodules remains a critical challenge in clinical practice. This study aimed to evaluate the diagnostic performance of computed tomography (CT) imaging features in distinguishing malignant from benign pulmonary nodules.

**Methods:**

A retrospective analysis was conducted on 200 patients with pulmonary nodules who underwent chest CT scanning and subsequent histopathological confirmation between January 2020 and December 2024. CT imaging features including nodule size, density, margin characteristics, internal characteristics, and relationship to adjacent structures were analyzed. To systematically evaluate the independent contribution of CT imaging features, multivariate logistic regression analysis was performed using a progressive modeling approach. Model stability was assessed using bootstrap internal validation (1,000 resamples) and verified by AIC-based backward stepwise selection. Receiver operating characteristic curve analysis was employed to assess the diagnostic performance of CT imaging features.

**Results:**

Among 200 cases, 88 (44.00%) were malignant and 112 (56.00%) were benign. Malignant nodules demonstrated significantly larger mean diameter (18.76 ± 8.52 mm vs. 12.85 ± 5.94 mm, *P* < 0.001), higher prevalence of spiculated margins (70.45% vs. 28.57%, *P* < 0.001), lobulation (67.05% vs. 33.04%, *P* < 0.001), and pleural indentation (54.55% vs. 22.32%, *P* < 0.001). Calcification was more common in benign nodules (43.75% vs. 12.50%, *P* < 0.001). After adjustment for clinical confounders, nodule size (OR = 1.095, *P* < 0.001), spiculated margin (OR = 4.523, *P* < 0.001), lobulation (OR = 2.845, *P* < 0.001), and calcification (OR = 0.185, *P* < 0.001) remained independent predictors of malignancy. AIC-based backward stepwise selection independently confirmed the same four CT features, supporting the robustness of variable selection. The integrated model incorporating four CT imaging features achieved sensitivity of 71.59%, specificity of 88.39%, and area under the curve of 0.870(optimism-corrected AUC = 0.845).

**Conclusion:**

Chest CT imaging features, particularly nodule size, spiculated margin, lobulation, and calcification patterns, are independent predictors of malignancy and demonstrate good diagnostic performance in differentiating malignant from benign pulmonary nodules. These findings provide preliminary evidence supporting the integration of structured CT feature analysis into clinical decision-making; however, external validation in independent cohorts is needed before clinical implementation.

## Background

Pulmonary nodules are frequently encountered in clinical practice, with increasing detection rates due to widespread adoption of screening programs and advanced imaging technologies [1]. A pulmonary nodule is defined radiologically as a rounded or irregular opacity measuring up to 30 mm in diameter, surrounded by aerated lung without associated atelectasis or lymphadenopathy [2]. The incidence of incidentally detected pulmonary nodules ranges from 10% to 60% in screening populations, presenting a significant diagnostic challenge for clinicians [3]. While the majority of pulmonary nodules are benign, timely identification of malignant lesions is crucial for optimal patient outcomes and survival rates.

Computed tomography (CT) remains the cornerstone imaging modality for evaluating pulmonary nodules, offering superior spatial resolution and detailed characterization of nodule morphology [4]. Various CT features have been investigated for their potential to differentiate malignant from benign nodules, including nodule size, density characteristics, margin features, growth patterns, and enhancement characteristics [5, 6]. However, substantial overlap exists in the imaging appearance of benign and malignant nodules, leading to diagnostic uncertainty and unnecessary invasive procedures [7, 8]. The Fleischner Society and other professional organizations have established guidelines for nodule management based primarily on size and morphology [9, 10]. Nevertheless, individualized risk stratification requires comprehensive evaluation of multiple imaging parameters. Recent studies have demonstrated that integrating various CT features can improve diagnostic accuracy, yet the optimal combination of features and their relative importance remain subjects of ongoing investigation [11, 12]. 

Despite advances in imaging technology, accurate differentiation between benign and malignant pulmonary nodules continues to pose challenges. False-positive findings lead to unnecessary biopsies, surgical procedures, and patient anxiety, while false-negative results may delay treatment of early-stage lung cancer. Therefore, identifying reliable CT imaging features that can distinguish malignant from benign nodules is of paramount clinical importance. The present study aimed to systematically evaluate the diagnostic performance of various chest CT imaging features in differentiating malignant from benign pulmonary nodules in a large cohort of patients with histopathologically confirmed diagnoses.

## Methods

### Study population

**Fig. 1:**
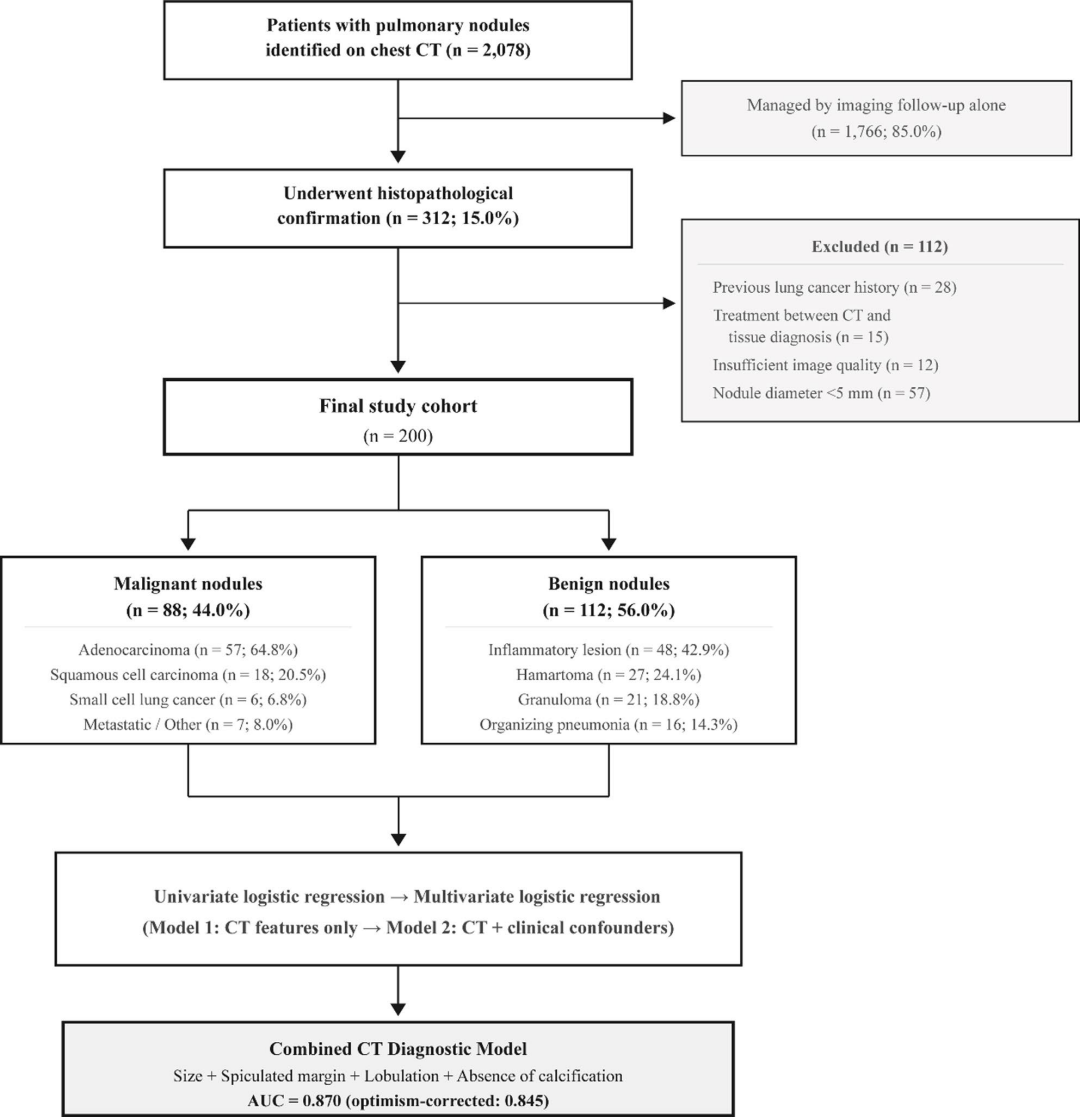
Study flowchart. From 2,078 patients with pulmonary nodules on chest CT, 312 (15.0%) underwent histopathological confirmation. After excluding 112 patients, 200 were included in the final analysis (88 malignant, 112 benign). CT, computed tomography; AUC, area under the curve

This retrospective study was conducted at Peking University Shenzhen Hospital following approval by the Institutional Review Board (IRB approval number: 2024 − 201). The requirement for written informed consent was waived given the retrospective design using anonymized data. Between January 2020 and December 2024, a total of 2,078 patients with pulmonary nodules detected on chest CT were identified from the institutional radiology database. Of these, 1,766 patients (85.0%) were managed by imaging follow-up alone. The remaining 312 patients (15.0%) underwent histopathological confirmation. After applying exclusion criteria, 112 patients were excluded: previous history of lung cancer (*n* = 28), treatment received between CT examination and tissue diagnosis (*n* = 15), insufficient image quality (*n* = 12), and nodule diameter < 5 mm (*n* = 57). The final study cohort comprised 200 patients. The patient selection process is illustrated in Fig. 1.

Inclusion criteria were: (1) patients aged 18 years or older; (2) presence of pulmonary nodule(s) detected on chest CT; (3) histopathological confirmation obtained through surgical resection, transthoracic needle biopsy, or bronchoscopic biopsy within three months of CT examination; (4) availability of complete clinical and imaging data. Exclusion criteria included: (1) previous history of lung cancer; (2) patients who received treatment between CT examination and tissue diagnosis; (3) insufficient image quality for evaluation; (4) nodules smaller than 5 mm in diameter.

Each patient contributed one target nodule for analysis. For patients with multiple pulmonary nodules, only the nodule that underwent histopathological confirmation was included. When multiple nodules were confirmed pathologically, the dominant nodule (defined as the largest or most morphologically suspicious lesion) was selected as the target lesion, ensuring one observation per patient to avoid intra-patient correlation.

### CT imaging protocol

All chest CT examinations were performed using multi-detector CT scanners (64-detector or higher). Scanning parameters included: tube voltage 120 kVp, automatic tube current modulation, slice thickness 1.0–1.25 mm, reconstruction interval 0.625–1.0 mm. Contrast-enhanced CT was performed in selected cases using intravenous injection of non-ionic contrast medium (1.5 mL/kg body weight) at a rate of 2.5-3.0 mL/s, followed by imaging at arterial phase (25–30 s) and venous phase (60–70 s). Of note, contrast-enhanced CT was performed based on clinical indication rather than a standardized protocol; its availability was therefore non-random and associated with larger nodule size and higher clinical suspicion (see Sensitivity Analyses).

### Image analysis

Two experienced radiologists (with 12 and 15 years of experience in thoracic imaging, respectively) independently reviewed all CT images, blinded to clinical information and histopathological results. Disagreements were resolved through consensus discussion. The following CT features were evaluated for each nodule:

#### Nodule size

Maximum diameter measured on axial images in lung window settings.

#### Nodule density

Classified as solid, part-solid (mixed ground-glass and solid components), or pure ground-glass opacity.

#### Margin characteristics

Smooth, lobulated, spiculated, or irregular.

#### Internal characteristics

Presence of calcification, cavitation, air bronchogram, or bubble-like lucency.

#### Relationship to adjacent structures

Pleural indentation, vascular convergence, and bronchus involvement.

#### Enhancement pattern

Measured as attenuation difference between pre-contrast and post-contrast images (when available), classified as no enhancement (< 15 HU), mild enhancement (15–25 HU), or moderate-to-marked enhancement (> 25 HU).

#### Location

Upper lobe, middle lobe, or lower lobe; peripheral or central.

To assess interobserver reproducibility, a post-hoc agreement analysis was performed on a random subset of 50 cases. The intraclass correlation coefficient (ICC) was used for continuous variables (nodule size), and Cohen’s kappa (κ) was used for categorical variables. Agreement was interpreted as: [13] poor (κ < 0.20), fair (κ = 0.21–0.40), moderate (κ = 0.41–0.60), substantial (κ = 0.61–0.80), and almost perfect (κ = 0.81–1.00).

### Clinical data collection

Clinical data including age, sex, smoking history (current/former vs. never smoker), family history of lung cancer, history of chronic obstructive pulmonary disease (COPD), diabetes mellitus, and hypertension were extracted from electronic medical records.

### Histopathological diagnosis

Histopathological diagnosis was established through surgical resection (*n* = 156), CT-guided transthoracic needle biopsy (*n* = 33), or bronchoscopic biopsy (*n* = 11). All specimens were processed according to standard protocols and examined by experienced pathologists. Malignant nodules were classified according to the 2021 World Health Organization classification of lung tumors [14]. Benign nodules were categorized as inflammatory lesions, hamartomas, granulomas, or other benign entities.

### Statistical analysis

Statistical analyses were performed using SPSS software version 26.0 (IBM Corp., Armonk, NY, USA). Continuous variables were expressed as mean ± standard deviation and compared using independent samples t-test. Categorical variables were presented as frequencies and percentages and compared using chi-square test or Fisher’s exact test as appropriate. Variables with *P* < 0.100 in univariate analysis were subsequently included in multivariate logistic regression analysis.

To systematically evaluate the independent contribution of CT imaging features while controlling for potential confounders, we employed a progressive modeling approach with two sequential models: Model 1: Target CT imaging variables only (including all CT features with *P* < 0.100 in univariate analysis: nodule size, part-solid density, spiculated margin, lobulation, smooth margin, calcification, air bronchogram, bubble-like lucency, pleural indentation, vascular convergence, and bronchus involvement). Model 2: Model 1 + clinical confounders (including age, smoking history, family history of lung cancer, and history of COPD). Based on the results of Model 2, CT features that retained independent significance (*P* < 0.001) were selected to construct a combined CT-based diagnostic model. The final regression coefficients and prediction equation are reported in the Results section.

Variance Inflation Factor (VIF) was calculated to assess multicollinearity, with VIF > 5 indicating significant collinearity. Model calibration was assessed using the Hosmer-Lemeshow goodness-of-fit test, with *P* > 0.05 indicating adequate calibration. Odds ratios (OR) with 95% confidence intervals (CI) were calculated. Receiver operating characteristic (ROC) curve analysis was conducted to evaluate the diagnostic performance of individual parameters and their combination. Pairwise AUC comparisons between the combined model and individual features were performed using the DeLong test. Area under the curve (AUC), sensitivity, specificity, positive predictive value (PPV), and negative predictive value (NPV) were calculated. The optimal probability cutoff for the combined model was determined using the Youden index (J = sensitivity + specificity − 1). For nodule size, the optimal diagnostic threshold was determined from the ROC curve using the same method. Bootstrap internal validation (1,000 resamples) was performed to estimate the optimism-corrected AUC and assess potential overfitting. The optimism was defined as the difference between the bootstrap-derived apparent AUC and the AUC obtained when each bootstrap model was applied to the original dataset. Two prespecified sensitivity analyses were performed to assess the robustness of the primary findings. To verify the consistency of variable selection, AIC-based backward stepwise elimination was applied starting from the full Model 2, allowing comparison between data-driven and significance-based predictor selection strategies. To evaluate the potential incremental contribution of contrast enhancement, a separate logistic regression analysis was conducted in the subset of 132 patients who underwent contrast-enhanced CT, incorporating moderate-to-marked enhancement as an additional candidate predictor alongside the four morphological features. A two-tailed P value < 0.050 was considered statistically significant.

## Results

### Patient characteristics and histopathological diagnosis

A total of 200 patients (116 males, 84 females) with mean age of 58.24 ± 12.18 years (range: 29–80 years) were included in the study. The demographic characteristics are summarized in Table 1. Among 200 pulmonary nodules, 88 (44.00%) were diagnosed as malignant and 112 (56.00%) as benign based on histopathological examination.

**Table 1:** Patient demographics and histopathological distribution

Characteristic	Overall (*n* = 200)	Malignant (*n* = 88)	Benign (*n* = 112)	*P* value
Age (years), mean ± SD	58.24 ± 12.18	61.35 ± 10.62	55.96 ± 12.87	< 0.001
**Sex**,** n (%)**				
Male	116 (58.00)	55 (62.50)	61 (54.46)	0.243
Female	84 (42.00)	33 (37.50)	51 (45.54)	
**Smoking history**,** n (%)**				
Current/former smoker	119 (59.50)	61 (69.32)	58 (51.79)	0.012
Never smoker	81 (40.50)	27 (30.68)	54 (48.21)	
**Family history**				
Yes	34 (17.00)	21 (23.86)	13 (11.61)	0.022
No	166 (83.00)	67 (76.14)	99 (88.39)	
**History of COPD**,** n (%)**				
Yes	42 (21.00)	25 (28.41)	17 (15.18)	0.023
No	158 (79.00)	63 (71.59)	95 (84.82)	
**Diabetes mellitus**,** n (%)**				
Yes	44 (22.00)	23 (26.14)	21 (18.75)	0.197
No	156 (78.00)	65 (73.86)	91 (81.25)	
**Hypertension**,** n (%)**				
Yes	86 (43.00)	42 (47.73)	44 (39.29)	0.213
No	114 (57.00)	46 (52.27)	68 (60.71)	
**Histopathological diagnosis**				
**Malignant nodules**	**88 (44.00)**			
Adenocarcinoma	57 (28.50)	57 (64.77)	-	
Squamous cell carcinoma	18 (9.00)	18 (20.45)	-	
Small cell lung cancer	6 (3.00)	6 (6.82)	-	
Metastatic tumor	4 (2.00)	4 (4.55)	-	
Others	3 (1.50)	3 (3.41)	-	
**Benign nodules**	**112 (56.00)**			
Inflammatory lesion	48 (24.00)	-	48 (42.86)	
Hamartoma	27 (13.50)	-	27 (24.11)	
Granuloma	21 (10.50)	-	21 (18.75)	
Organizing pneumonia	16 (8.00)	-	16 (14.29)	
Solitary nodule, n (%)	118 (59.00)	50 (56.82)	68 (60.71)	0.576

Note: Data are presented as mean ± standard deviation for continuous variables and n (%) for categorical variables. P values were calculated using independent samples t-test for continuous variables and chi-square test or Fisher’s exact test for categorical variables. SD, standard deviation; COPD, chronic obstructive pulmonary disease

### CT imaging features

The CT imaging characteristics of malignant and benign nodules are compared in Table 2. Malignant nodules showed significantly larger mean diameter compared to benign nodules (18.76 ± 8.52 mm vs. 12.85 ± 5.94 mm, *P* < 0.001). Regarding margin characteristics, spiculated margins were observed in 70.45% of malignant nodules compared to 28.57% of benign nodules (*P* < 0.001). Lobulation was present in 67.05% of malignant nodules versus 33.04% of benign nodules (*P* < 0.001). In contrast, smooth margins were more common in benign nodules (56.25% vs. 10.23%, *P* < 0.001). Internal calcification was significantly more frequent in benign nodules (43.75%) compared to malignant nodules (12.50%, *P* < 0.001). Pleural indentation was observed in 54.55% of malignant nodules versus 22.32% of benign nodules (*P* < 0.001). Vascular convergence was also more common in malignant nodules (48.86% vs. 25.89%, *P* = 0.001). Among 132 patients who underwent contrast-enhanced CT, moderate-to-marked enhancement was more frequently seen in malignant nodules (51.61%) compared to benign nodules (25.71%, *P* = 0.005).

Interobserver agreement for CT feature assessment was evaluated in a random subset of 50 cases. Excellent agreement was observed for nodule size (ICC = 0.95) and calcification (κ = 0.87). Substantial agreement was found for nodule density (κ = 0.82), smooth margin (κ = 0.72), and air bronchogram (κ = 0.65). Moderate agreement was noted for spiculated margin (κ = 0.61), lobulation (κ = 0.58), and vascular convergence (κ = 0.56). The four predictors in the final combined model demonstrated moderate to excellent agreement (κ/ICC range: 0.58–0.95).

**Table 2:** Comparison of CT imaging features between malignant and benign nodules

CT Feature	Malignant (*n* = 88)	Benign (*n* = 112)	*P* value
**Size (mm)**,** mean ± SD**	18.76 ± 8.52	12.85 ± 5.94	< 0.001
**Density**,** n (%)**			
Solid	67 (76.14)	97 (86.61)	0.004
Part-solid	16 (18.18)	10 (8.93)	
Ground-glass	5 (5.68)	5 (4.46)	
**Margin characteristics**,** n (%)**			
Spiculated margin	62 (70.45)	32 (28.57)	< 0.001
Lobulation	59 (67.05)	37 (33.04)	< 0.001
Smooth margin	9 (10.23)	63 (56.25)	< 0.001
**Internal characteristics**,** n (%)**			
Calcification	11 (12.50)	49 (43.75)	< 0.001
Cavitation	13 (14.77)	12 (10.71)	0.378
Air bronchogram	24 (27.27)	19 (16.96)	0.075
Bubble-like lucency	21 (23.86)	15 (13.39)	0.054
**Adjacent structure involvement**,** n (%)**			
Pleural indentation	48 (54.55)	25 (22.32)	< 0.001
Vascular convergence	43 (48.86)	29 (25.89)	0.001
Bronchus involvement	18 (20.45)	13 (11.61)	0.084
**Location**,** n (%)**			
Upper lobe	51 (57.95)	59 (52.68)	0.683
Middle lobe	9 (10.23)	15 (13.39)	
Lower lobe	28 (31.82)	38 (33.93)	
**Enhancement pattern (*n* = 132)**,** n (%)**			
No enhancement	5 (8.06)	17 (24.29)	0.005
Mild enhancement	25 (40.32)	35 (50.00)	
Moderate-marked enhancement	32 (51.61)	18 (25.71)	

Note: Data are presented as mean ± standard deviation for continuous variables and n (%) for categorical variables. P values were calculated using independent samples t-test for continuous variables and chi-square test or Fisher’s exact test for categorical variables. For nodule density, solid nodules served as the reference category. Enhancement pattern was evaluated in 132 patients who underwent contrast-enhanced CT examination (62 malignant, 70 benign). SD, standard deviation; CT, computed tomography; HU, Hounsfield unit

### Univariate analysis

Univariate logistic regression analysis identified multiple clinical and CT imaging features significantly associated with malignancy (Table 3).

**Table 3:** Univariate logistic regression analysis

Variable	Odds Ratio	95% CI	*P* value
Clinical characteristics			
Age (per year increase)	1.045	1.022–1.068	< 0.001
Sex (male vs. female)	1.392	0.812–2.387	0.228
Smoking history (yes vs. no)	2.112	1.192–3.741	0.010
Family history (yes vs. no)	2.410	1.121–5.183	0.024
COPD (yes vs. no)	2.187	1.109–4.313	0.024
Diabetes mellitus (yes vs. no)	1.533	0.794–2.960	0.203
Hypertension (yes vs. no)	1.412	0.822–2.426	0.212
**CT imaging features**			
Nodule size (per mm increase)	1.092	1.053–1.133	< 0.001
Part-solid density (vs. Solid)	2.320	0.972–5.540	0.058
Ground-glass density (vs. Solid)	1.448	0.393–5.337	0.578
Spiculated margin (yes vs. no)	5.875	3.276–10.535	< 0.001
Lobulation (yes vs. no)	4.091	2.321–7.209	< 0.001
Smooth margin (yes vs. no)	0.086	0.039–0.190	< 0.001
Calcification (yes vs. no)	0.188	0.091–0.388	< 0.001
Cavitation (yes vs. no)	1.447	0.623–3.361	0.392
Air bronchogram (yes vs. no)	1.838	0.945–3.576	0.073
Bubble-like lucency (yes vs. no)	2.012	0.972–4.164	0.060
Pleural indentation (yes vs. no)	4.102	2.254–7.463	< 0.001
Vascular convergence (yes vs. no)	2.711	1.526–4.815	0.001
Bronchus involvement (yes vs. no)	1.962	0.904–4.257	0.089

Note: Data are presented as odds ratio (OR) with 95% confidence interval (CI). P values < 0.05 were considered statistically significant. For nodule density, solid nodules served as the reference category. COPD, chronic obstructive pulmonary disease; CI, confidence interval; OR, odds ratio

### Univariate and multivariate analysis

#### Multicollinearity assessment

Prior to multivariate analysis, VIF was calculated for variables showing statistical significance (*P* < 0.100) in univariate analysis to assess multicollinearity. The results are presented in Table 4. Most VIF values were below 3.0, well under the commonly accepted threshold of 5.0.

**Table 4:** Variance inflation factor (VIF) analysis

Variable	VIF
CT Imaging Features	
Nodule size (per mm)	1.89
Part-solid density	1.34
Spiculated margin	4.52
Lobulation	3.89
Smooth margin	4.45
Calcification	2.15
Air bronchogram	1.67
Bubble-like lucency	1.52
Pleural indentation	2.78
Vascular convergence	2.34
Bronchus involvement	1.45
**Clinical Parameters**	
Age (per year)	1.24
Smoking history	1.56
Family history	1.23
COPD	1.38

Note: Variance Inflation Factor (VIF) was calculated to assess multicollinearity among variables with *P* < 0.100 in univariate analysis. VIF values > 5.0 indicate significant multicollinearity. All VIF values in this analysis were below 5.0, indicating acceptable levels of collinearity. VIF, variance inflation factor; COPD, chronic obstructive pulmonary disease

#### Multivariate logistic regression analysis

To address potential confounding factors and systematically evaluate the independent contribution of CT imaging features, we performed multivariate logistic regression analysis using two sequential models. Model 1 included only CT imaging variables with *P* < 0.100 in univariate analysis to establish their baseline associations with malignancy. Model 2 additionally incorporated clinical confounders (age, smoking history, family history, and COPD) to determine whether CT features retained their predictive value after adjustment for patient characteristics. The Hosmer-Lemeshow test indicated good model fit for both models (Model 1: χ²=6.234, df = 8, *P* = 0.621; Model 2: χ²=7.156, df = 8, *P* = 0.519), suggesting that predicted probabilities matched observed outcomes across risk strata. In Model 1, six CT imaging features were significantly associated with malignancy: nodule size (OR = 1.108, 95% CI: 1.051–1.168, *P* < 0.001), spiculated margin (OR = 4.965, 95% CI: 2.401–10.268, *P* < 0.001), lobulation (OR = 3.187, 95% CI: 1.612–6.301, *P* < 0.001), calcification (OR = 0.172, 95% CI: 0.076–0.389, *P* < 0.001), smooth margin (OR = 0.234, 95% CI: 0.089–0.615, *P* < 0.05), and pleural indentation (OR = 2.105, 95% CI: 1.001–4.428, *P* < 0.05). After adjustment for clinical confounders in Model 2, four CT imaging features remained independently and strongly associated with malignancy at *P* < 0.001 level: nodule size (OR = 1.095, 95% CI: 1.042–1.151), spiculated margin (OR = 4.523, 95% CI: 2.156–9.487), lobulation (OR = 2.845, 95% CI: 1.423–5.688), and calcification (OR = 0.185, 95% CI: 0.081–0.423). Among clinical parameters in Model 2, age showed a strong independent association with malignancy (OR = 1.038, 95% CI: 1.014–1.063, *P* < 0.001), and smoking history was also significantly associated (OR = 1.856, 95% CI: 1.015–3.394, *P* < 0.05). The detailed results of multivariate logistic regression analysis are presented in Table 5.

**Table 5:** Multivariate logistic regression analysis for predicting malignancy

Variable	Model 1	Model 2
	OR (95% CI)	OR (95% CI)
**CT Imaging Features**		
Nodule size (per mm)	1.108 (1.051–1.168)*	1.095 (1.042–1.151)*
Part-solid density (vs. Solid)	1.456 (0.567–3.745)	1.234 (0.478–3.189)
Spiculated margin (yes vs. no)	4.965(2.401–10.268)*	4.523 (2.156–9.487)*
Lobulation (yes vs. no)	3.187 (1.612–6.301)*	2.845 (1.423–5.688)*
Smooth margin (yes vs. no)	0.234 (0.089–0.615)†	0.267 (0.098–1.001)
Calcification (yes vs. no)	0.172 (0.076–0.389)*	0.185 (0.081–0.423)*
Air bronchogram (yes vs. no)	1.523 (0.745–3.115)	1.412 (0.678–2.941)
Bubble-like lucency (yes vs. no)	1.678 (0.789–3.567)	1.534 (0.712–3.304)
Pleural indentation (yes vs. no)	2.105 (1.001–4.428)†	1.923 (0.886–4.174)
Vascular convergence (yes vs. no)	1.845 (0.891–3.821)	1.756 (0.834–3.698)
Bronchus involvement (yes vs. no)	1.534 (0.678–3.468)	1.423 (0.623–3.248)
**Clinical Parameters**		
Age (per year)	-	1.038 (1.014–1.063)*
Smoking history (yes vs. no)	-	1.856 (1.015–3.394)†
Family history (yes vs. no)	-	1.734 (0.812–3.705)
COPD (yes vs. no)	-	1.623 (0.756–3.485)

Note: Data are presented as odds ratio with 95% confidence interval. Model 1 included only CT imaging variables with *P* < 0.100 in univariate analysis. Model 2 additionally incorporated clinical confounders (age, smoking history, family history of lung cancer, and history of COPD). For nodule density, solid nodules served as the reference category. Hosmer-Lemeshow goodness-of-fit test indicated adequate model calibration for both models (Model 1: χ²=6.234, df = 8, *P* = 0.621; Model 2: χ²=7.156, df = 8, *P* = 0.519). *P < 0.001; †*P* < 0.05. CI, confidence interval; COPD, chronic obstructive pulmonary disease; CT, computed tomography; NS, not statistically significant; OR, odds ratio

### Diagnostic performance

**Fig. 2:**
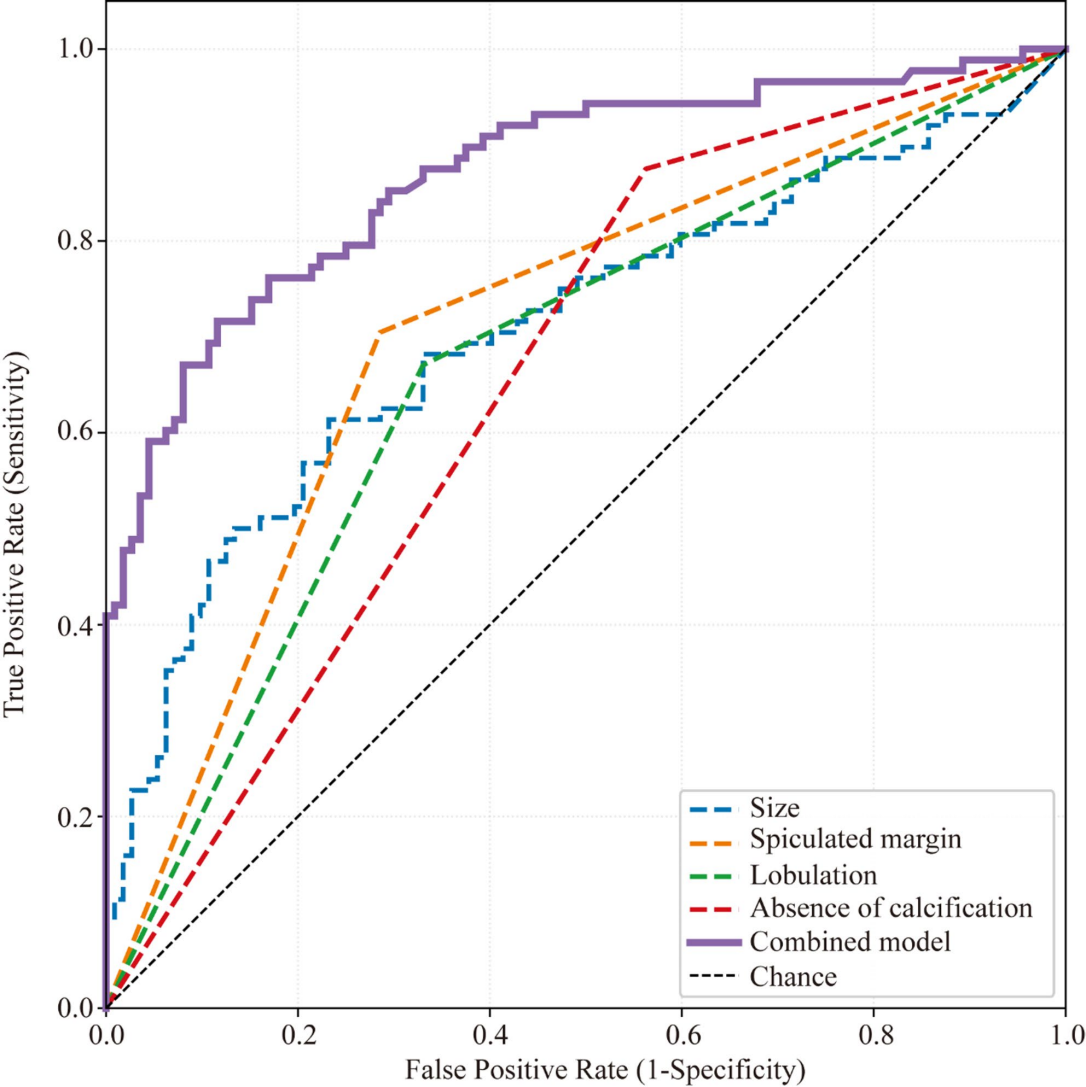
Diagnostic performance of CT imaging features for differentiating malignant from benign pulmonary nodules

The diagnostic performance of individual CT features and the combined multivariate model is presented in Table 6; Fig. 2. The combined model incorporated four CT features that remained independently significant in Model 2 after adjusting for clinical confounders: nodule size, spiculated margin, lobulation, and absence of calcification. The final logistic regression equation was: logit(P) = − 2.347 + 0.091 × size (mm) + 1.509 × spiculated + 1.045 × lobulation − 1.687 × calcification. ROC curve analysis showed that the combined model achieved an AUC of 0.870 (95% CI: 0.818–0.922), which was significantly higher than any individual CT feature (DeLong test: vs. spiculated margin [AUC = 0.709], *P* < 0.001; vs. nodule size [AUC = 0.707], *P* < 0.001; vs. lobulation [AUC = 0.670], *P* < 0.001; vs. absence of calcification [AUC = 0.656], *P* < 0.001). At the optimal probability threshold of 0.478, determined by the Youden index (maximizing sensitivity + specificity − 1), the combined model yielded a sensitivity of 71.59%, specificity of 88.39%, positive predictive value of 82.89%, negative predictive value of 79.84%, and overall accuracy of 81.00%. Bootstrap internal validation (1,000 resamples) yielded an optimism-corrected AUC of 0.845 (optimism = 0.025) for the combined model, indicating minimal overfitting. For individual binary predictors, optimism correction had negligible effect (optimism < 0.001), consistent with the absence of overfitting capacity in single-variable models. The optimism-corrected AUC for nodule size (continuous variable) was 0.704.

To address whether clinical variables provide additional diagnostic value beyond CT features, we compared three models: a clinical-only model incorporating age and smoking history (AUC = 0.690), the CT-only model incorporating the four significant CT features (AUC = 0.870), and a combined clinicoradiologic model incorporating all six significant predictors from Model 2 (AUC = 0.879). The CT-only model significantly outperformed the clinical-only model (DeLong test, *P* < 0.001). The addition of clinical variables to CT features did not significantly improve discrimination (DeLong test, *P* = 0.198), confirming that the four CT features captured the predominant diagnostic information.

**Table 6:** Diagnostic performance of CT features for differentiating malignant from benign nodules

Feature/Model	Sensitivity (%)	Specificity (%)	PPV (%)	NPV (%)	Accuracy (%)	AUC	Optimism-corrected AUC
Size	61.36	76.79	67.50	71.67	70.00	0.707	0.704
Spiculated margin	70.45	71.43	65.96	75.47	71.00	0.709	0.709
Lobulation	67.05	66.96	61.46	72.12	67.00	0.670	0.670
Absence of calcification	87.50	43.75	55.00	81.67	63.00	0.656	0.656
Combined model	71.59	88.39	82.89	79.84	81.00	0.870	0.845

Note: The combined model incorporated nodule size, spiculated margin, lobulation, and calcification, selected as independently significant CT features after adjusting for clinical confounders in Model 2. The final prediction equation was: logit(P) = − 2.347 + 0.091 × size (mm) + 1.509 × spiculated + 1.045 × lobulation − 1.687 × calcification. The optimal probability threshold for the combined model (0.478) was determined using the Youden index (maximizing sensitivity + specificity − 1). Individual feature performance was evaluated at presence versus absence for categorical features; for nodule size, the optimal threshold was determined by ROC analysis (16.51 mm). PPV, positive predictive value; NPV, negative predictive value; AUC, area under the curve; CI, confidence interval; CT, computed tomography

### Sensitivity analyses

#### Variable selection verification

AIC-based backward stepwise selection starting from the full Model 2 (15 predictors) sequentially eliminated bronchus involvement (ΔAIC = − 1.8), bubble-like lucency (ΔAIC = − 1.5), part-solid density (ΔAIC = − 1.3), vascular convergence (ΔAIC = − 1.1), COPD (ΔAIC = − 0.9), family history (ΔAIC = − 0.7), air bronchogram (ΔAIC = − 0.4), pleural indentation (ΔAIC = − 0.2), and smooth margin (ΔAIC = − 0.1). The final AIC-selected model retained six predictors: nodule size (OR = 1.089, *P* < 0.001), spiculated margin (OR = 4.312, *P* < 0.001), lobulation (OR = 2.756, *P* = 0.002), calcification (OR = 0.192, *P* < 0.001), age (OR = 1.035, *P* = 0.003), and smoking history (OR = 1.742, *P* = 0.048), with a minimum AIC of 187.3 compared with 194.8 for the full model. Notably, the four CT features identified by the significance-based approach were retained in the AIC-selected model, confirming the robustness of the primary variable selection.

#### Enhancement pattern analysis

In the subset of 132 patients with contrast-enhanced CT data (62 malignant, 70 benign), the four-variable CT model achieved an AUC of 0.876 (95% CI: 0.814–0.938). Adding moderate-to-marked enhancement as a fifth predictor yielded an AUC of 0.889 (95% CI: 0.831–0.947); the improvement was not statistically significant (DeLong test, *P* = 0.312). In the expanded model, enhancement did not achieve independent significance (OR = 1.78, 95% CI: 0.82–3.87, *P* = 0.143), while the four original predictors maintained significance with similar effect sizes (nodule size OR = 1.087, spiculated margin OR = 4.256, lobulation OR = 2.689, calcification OR = 0.198; all *P* < 0.005). Enhancement data were available in 66.0% of the cohort; patients who underwent contrast-enhanced CT had larger nodules (mean 17.2 ± 8.1 mm vs. 12.4 ± 5.8 mm, *P* = 0.001) and higher clinical suspicion of malignancy, indicating non-random missingness. These findings support the exclusion of enhancement from the primary model.

### Representative cases

**Fig. 3:**
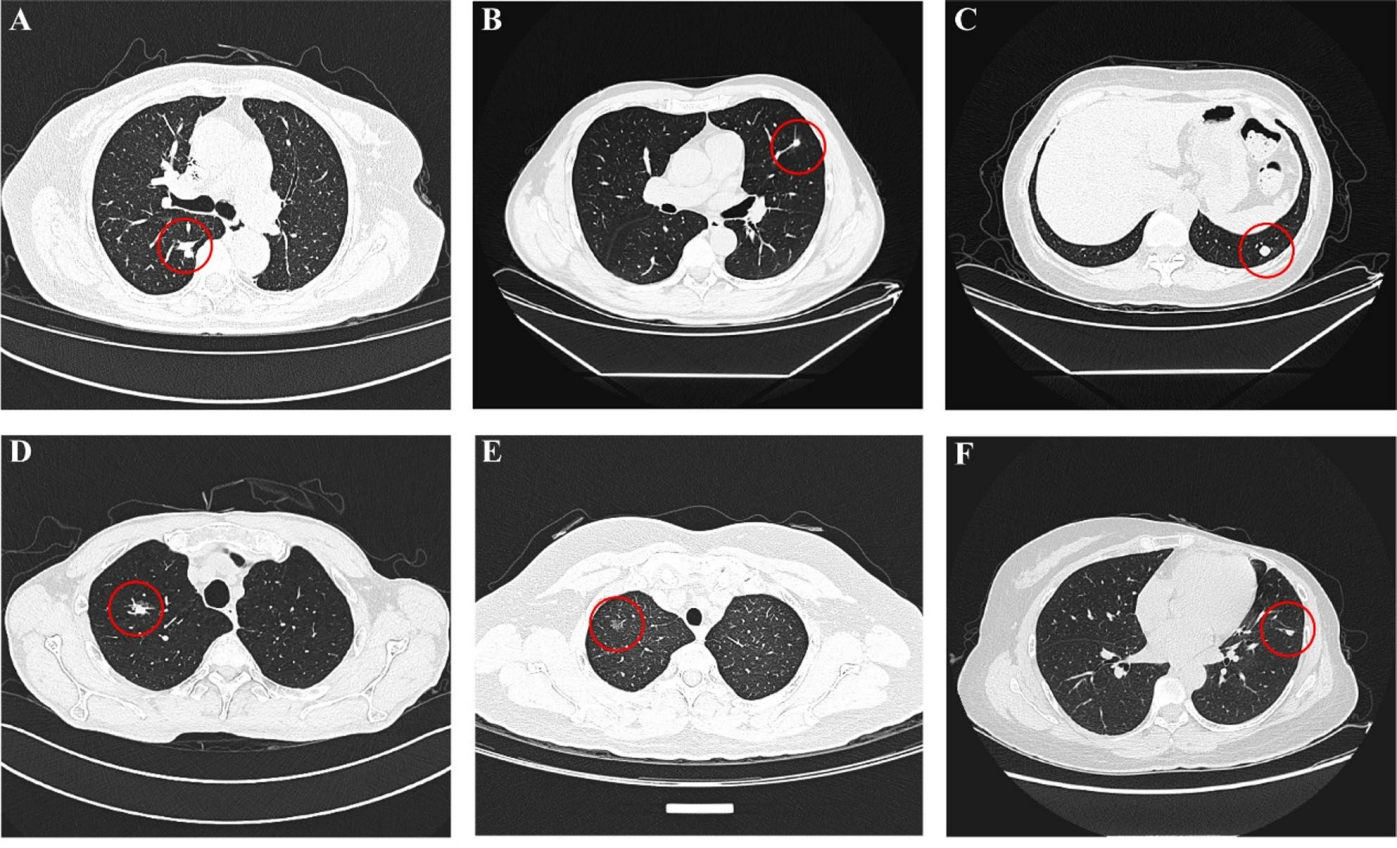
Representative CT images of pulmonary nodules demonstrating key imaging features. **Case A**: Patient in their 70s with chronic granulomatous inflammation, showing a nodule in the posterior segment of the right upper lobe with irregular morphology, lobulated margins, and pleural indentation sign, demonstrating imaging features mimicking malignancy. **Case B**: Patient in their 40s with an inflammatory lesion, displaying a solid nodule measuring approximately 7 mm × 6 mm in the superior lingular segment of the left upper lobe with lobulation, spiculated margins, and vascular convergence sign, also showing imaging characteristics suggestive of malignancy. **Case C**: Patient in their 50s with hamartoma, showing a round nodule with smooth margins and no internal calcification in the posterior basal segment of the left lower lobe, demonstrating typical benign imaging features. **Case D**: Patient in their 60s with invasive pulmonary adenocarcinoma, demonstrating a part-solid (mixed ground-glass) nodule in the apical segment of the right upper lobe with irregular morphology, spiculated margins, and solid component exceeding 5 mm, showing definite imaging features of malignancy. **Case E**: Patient in their 60s with minimally invasive adenocarcinoma, presenting a ground-glass nodule in the apical segment of the right upper lobe with ill-defined margins, measuring approximately 9 mm in maximum diameter, with small vessels penetrating into the lesion, demonstrating features consistent with malignancy. **Case F**: Patient in their 40s with metastatic carcinoma and known history of breast cancer, showing a small nodule adjacent to the oblique fissure pleura in the left lower lobe with well-defined margins, closely abutting the interlobar pleura, without lobulation or spiculation; despite benign imaging appearance, metastasis cannot be excluded given the patient’s oncologic history

Representative CT images of six cases (Fig. 3) demonstrate the spectrum of imaging appearances between benign lesions (granuloma, inflammatory nodule, hamartoma) and malignant tumors (adenocarcinoma, metastasis). These cases highlight the spectrum of imaging appearances from benign to malignant lesions, including instances where benign lesions (Cases A and B) can mimic malignancy and where malignant lesions (Case F) may appear benign, underscoring the importance of integrating multiple CT features with clinical context for accurate diagnosis.

## Discussion

This retrospective study systematically evaluated the diagnostic performance of CT imaging features in differentiating malignant from benign pulmonary nodules in a cohort of 200 patients with histopathologically confirmed diagnoses. Our findings demonstrate that nodule size, spiculated margin, lobulation, and calcification are independent predictors of malignancy, with the combined multivariate model achieving good diagnostic performance (AUC 0.870, sensitivity 71.59%, specificity 88.39%). These results provide preliminary evidence supporting the integration of comprehensive CT feature analysis into clinical decision-making for pulmonary nodule management.

The independent association between nodule size and malignancy risk has been well established in previous studies and forms the foundation of current management guidelines [9, 15]. Nodule size is nevertheless a complex biomarker that reflects cumulative growth dynamics rather than a single biological attribute, and its relationship with malignancy is confounded by growth kinetics, histological subtype, and detection bias [16]. Moreover, size alone demonstrated limited specificity, underscoring the necessity of incorporating additional morphological features for accurate diagnosis [17]. 

Spiculated margin emerged as the strongest morphological predictor of malignancy, with an adjusted odds ratio of 4.523. This radiological feature corresponds pathologically to malignant cells infiltrating along bronchovascular bundles, interlobular septa, and lymphatic channels, a pattern characteristic of lepidic spread in pulmonary adenocarcinoma [18]. This process is termed “lepidic spread” in adenocarcinoma [19, 20]. This infiltrative architecture contrasts sharply with the expansile growth pattern typical of inflammatory lesions, which tend to compress rather than invade adjacent structures [21]. The presence of spiculation in 70.45% of malignant nodules compared to 28.57% of benign lesions in our study highlights its high specificity for malignancy. Nevertheless, the subjective nature of spiculation assessment introduces inherent measurement variability that must be carefully considered when applying this finding in clinical practice [22]. 

Lobulation, characterized by undulating or scalloped nodule margins, emerged as an independent predictor of malignancy in our analysis with an adjusted odds ratio of 2.845. This morphological feature reflects the heterogeneous growth pattern of malignant tumors, where different portions of the nodule expand at varying rates due to regional differences in blood supply, cellular proliferation, and stromal resistance [23, 24]. However, the diagnostic specificity of lobulation is substantially lower than spiculation, as similar morphology can occur in benign conditions including hamartomas and granulomatous diseases [20]. This limited specificity poses diagnostic challenges, as granulomatous nodules exhibiting lobulation and even spiculation are sometimes misdiagnosed as malignant lesions, necessitating pathological confirmation [25]. Despite these limitations, lobulation retained independent predictive value in our multivariate model after adjustment for other morphological features and clinical factors, suggesting it provides complementary diagnostic information beyond spiculation alone. The moderate correlation between lobulation and spiculation (VIF = 3.89) reflects their shared pathophysiological basis in irregular tumor growth. However, their distinct patterns, namely smooth undulations versus radiating spicules, capture different aspects of tumor-parenchyma interaction.

The protective association of calcification requires nuanced interpretation beyond simple presence-absence dichotomization. Pathologically, different calcification patterns reflect distinct biological processes: central or laminated calcification typically indicates granulomatous inflammation with dystrophic mineralization, while popcorn-like calcification suggests chondroid differentiation in hamartomas [26]. However, malignant nodules can occasionally exhibit eccentric or punctate calcification, particularly in cases of carcinoma arising within pre-existing fibrotic scars or engulfing granulomatous foci [26]. Our analysis did not subcategorize calcification patterns due to sample size limitations, and this aggregation may obscure important distinctions. The strong protective effect observed (OR = 0.185) likely reflects predominance of typical benign patterns in our cohort, but the possibility of false reassurance from atypical malignant calcification patterns warrants clinical vigilance.

The progressive modeling approach employed in this study provides distinct methodological advantages. By analyzing CT features alone in Model 1 before adding clinical confounders in Model 2, we systematically delineated the independent contribution of imaging characteristics beyond what can be obtained from clinical history. The retention of all four key features as significant predictors after adjustment for age, smoking history, family history, and COPD demonstrates the robust diagnostic value of CT imaging. This finding addresses a fundamental question in radiological research, namely whether imaging features provide incremental diagnostic information independent of clinical factors. The slight reduction in odds ratios after adjustment reflects expected overlap between imaging and clinical risk factors, yet the maintained statistical significance confirms substantial independent predictive value. This methodological rigor strengthens the evidence base for integrating comprehensive CT feature analysis into clinical decision-making algorithms.

In our study, the integration of multiple CT imaging features, specifically nodule size, spiculated margin, lobulation, and calcification, demonstrated significantly superior diagnostic performance (AUC 0.870) compared to any single parameter. Direct numerical comparisons of diagnostic performance metrics require substantial caution due to marked heterogeneity in study populations, outcome definitions, and analytical approaches [27]. Studies including screening populations with predominantly small nodules differ fundamentally from surgical cohorts enriched for larger, more suspicious lesions [28, 29]. Additionally, variations in histopathological confirmation rates introduce verification bias of varying magnitude [30]. These heterogeneities render our model fundamentally distinct from screening-oriented, low-confirmation cohorts such as Brock and VA in terms of baseline malignancy risk and imaging feature spectrum [31, 32]. 

Furthermore, the clinical translation of our findings necessitates careful consideration of implementation context. While integrated risk prediction incorporating multiple CT features offers improved diagnostic accuracy over single-parameter assessment, real-world application confronts challenges of interobserver variability, particularly for subjective features like spiculation and lobulation. Training and standardization initiatives, potentially augmented by computer-aided detection systems, become essential for maintaining consistency across interpreters and institutions. Structured reporting templates incorporating these features could facilitate systematic documentation and clinical decision support. However, the risk of algorithmic over-reliance deserves attention. CT feature assessment should inform rather than replace clinical judgment integrating patient-specific factors and preferences [33]. The balance between standardization and individualization represents an ongoing tension in evidence-based imaging [34]. 

The sensitivity analyses strengthen the internal validity of our findings. The concordance between significance-based and AIC-based variable selection confirms that the four CT predictors were not artifacts of a single analytical strategy. The enhancement sensitivity analysis demonstrated that contrast enhancement, although associated with malignancy in univariate analysis, did not contribute independent predictive value beyond morphological features; however, this analysis was limited by the non-random availability of contrast-enhanced data.

Our study has several important limitations beyond those previously noted. The single-center design limits assessment of generalizability across different patient populations, practice patterns, and imaging equipment. The retrospective nature precludes systematic evaluation of temporal stability, including whether initial CT features reliably predict behavior during surveillance or whether evolutionary changes provide additional diagnostic information. Several methodological considerations warrant discussion. The requirement for histopathological confirmation introduces inherent selection and verification bias. Of 2,078 patients with pulmonary nodules, only 312 (15.0%) underwent biopsy or resection, and the final cohort was enriched with surgical cases (78.0% surgical resection). Consequently, the study population likely represents a higher-risk subset with larger and more morphologically suspicious nodules compared to the general population of incidental pulmonary nodules. The reported AUC of 0.870 may therefore overestimate performance in unselected screening populations, where the prevalence and spectrum of malignancy differ substantially. A related limitation concerns the absence of longitudinal imaging data. Prior CT examinations were not available for the majority of patients, precluding assessment of interval growth, which is a well-established predictor of malignancy. Future studies incorporating serial imaging data may improve diagnostic accuracy. Moreover, although 41% of patients had multiple nodules, the primary regression models did not stratify by nodule multiplicity. It remains possible that individual predictor effects vary between solitary and multiple nodule populations, and dedicated analyses in these subgroups are warranted.

From a measurement perspective, smoking history was recorded as a dichotomous variable (current/former vs. never) without quantification of pack-years. This crude categorization may underestimate the true confounding effect of smoking intensity on malignancy risk. Similarly, interobserver agreement for spiculated margin (κ = 0.61) and lobulation (κ = 0.58), two of the four predictors in the final model, reached only moderate levels. This measurement variability may attenuate the observed associations and limit reproducibility across different interpreters and institutions. Regarding model stability, the events-per-variable ratio was 8.0 for Model 1 and 5.9 for Model 2, below the traditional threshold of 10 but within the acceptable range demonstrated by simulation studies [35]. The final combined model (EPV = 22.0) and the modest bootstrap optimism (0.025) mitigate overfitting concerns, though the full models should be interpreted with appropriate caution. Enhancement pattern was excluded from the primary regression model because contrast-enhanced CT data were available in only 66.0% of patients, and this availability was non-random, being associated with larger nodule size and higher clinical suspicion. Sensitivity analysis in the contrast-enhanced subset showed no independent contribution of enhancement, but the non-random missingness limits the generalizability of this finding. Finally, the model was developed and evaluated using the same dataset without an independent validation cohort. Although bootstrap internal validation suggested modest optimism (optimism-corrected AUC = 0.845), external validation in geographically and demographically distinct populations is essential before clinical adoption.

The absence of cost-effectiveness analysis leaves uncertain whether comprehensive CT feature assessment provides sufficient incremental value to justify additional radiologist time and specialized training compared to simplified algorithms focused on size thresholds. Additionally, our analysis does not address the growing recognition of radiological-pathological discordance, where nodules exhibiting benign imaging characteristics occasionally harbor occult malignancy, particularly in certain histological subtypes. These false-reassurance scenarios, though uncommon, carry significant clinical consequences requiring explicit acknowledgment in patient counseling and management planning. Future research should focus on longitudinal studies evaluating how CT features predict growth rates and time to diagnosis, which could refine surveillance interval recommendations. Investigation of feature interactions with patient-level factors such as genetic risk profiles could enable more personalized risk assessment. Development of validated artificial intelligence systems for automated feature extraction may reduce interobserver variability while improving efficiency. Application of our findings to special populations including never-smokers and patients with pre-existing lung disease requires dedicated investigation, as these groups may exhibit different feature distributions. Integration of CT features with complementary diagnostic modalities such as FDG-PET and circulating biomarkers represents another promising direction for comprehensive multidimensional risk prediction.

## Conclusion

Our study demonstrates that comprehensive evaluation of CT imaging features, particularly nodule size, spiculated margins, lobulation, and calcification patterns, provides good diagnostic performance in differentiating malignant from benign pulmonary nodules. The progressive modeling approach employed in this study, with systematic adjustment for clinical confounders, provides preliminary evidence for the independent contribution of CT imaging features to malignancy prediction. Sensitivity analyses using AIC-based variable selection and enhancement pattern evaluation corroborated the robustness of the primary findings. These findings support the integration of structured CT feature analysis into clinical decision-making algorithms for pulmonary nodule management, potentially improving diagnostic accuracy while reducing unnecessary invasive procedures. However, the model’s performance may be overestimated due to verification bias inherent in the study design. Future multicenter prospective studies with external validation cohorts are needed to confirm these findings and facilitate widespread clinical implementation.

## Data Availability

The datasets used and analyzed during the current study are available from the corresponding author on reasonable request.

## Abbreviations

AUC: Area Under the Curve
BAC: Bronchioloalveolar Carcinoma
CI: Confidence Interval
COPD: Chronic Obstructive Pulmonary Disease
CT: Computed Tomography
FDG-PET: Fluorodeoxyglucose Positron Emission Tomography
HRCT: High-Resolution Computed Tomography
HU: Hounsfield Unit
IRB: Institutional Review Board
MSA-Net: Multiple Self-Attention Network
NPV: Negative Predictive Value
NS: Not Statistically Significant
OR: Odds Ratio
PPV: Positive Predictive Value
ROC: Receiver Operating Characteristic
SD: Standard Deviation
SPN: Solitary Pulmonary Nodule
VA: Veterans Affairs
VIF: Variance Inflation Factor
AIC: Akaike Information Criterion
EPV: Events Per Variable
ICC: Intraclass Correlation Coefficient
